# Narcotic offences and drug use disorders among young refugees in Norway

**DOI:** 10.1177/14034948231201895

**Published:** 2023-10-05

**Authors:** Ryan T. Europa, Ketil Eide, Anders Hjern, Helio Manhica, Andrea Dunlavy

**Affiliations:** 1 Department of Health, Social and Welfare Studies, University of South-Eastern Norway, Norway; 2 Department of Medicine, Clinical Epidemiology, Karolinska Institutet, Sweden; 3 Centre for Health Equity Studies, Department of Public Health Sciences, Stockholm University, Sweden; 4 Department of Global Public Health, Karolinska Institutet, Sweden

**Keywords:** Young refugees, immigrant health, ethnic minorities, prejudicial policing

## Abstract

**Aims:**

We examined the patterns of healthcare utilisation for drug use disorders (DUDs) and charges related to narcotics among young refugees in Norway considering the role of sex, country of origin and condition of arrival (accompanied versus unaccompanied minors).

**Methods:**

Based on national registers, sex-stratified Cox regression models were used to estimate hazard ratios to assess the risk of being charged with a narcotics offence and the use of healthcare services related to DUDs. The sample consisted of 15,068 young refugees and 573,241 young Norwegians born in Norway to two Norwegian-born parents. All of the young people in the sample were born between 1983 and 1994. The follow-up period was from January 2008 to December 2015.

**Results:**

Compared with their Norwegian peers, both male and female refugees showed either a similar or lower risk of receiving healthcare for DUDs. However, male refugees showed an increased risk of being charged with a narcotic offence, except those from Afghanistan and the former Yugoslavia. Accompanied male refugees were at a higher risk of being charged, while unaccompanied male refugees showed a lower risk.

**Conclusions:**

**Young male refugees generally had a higher risk of being charged for narcotic offences while showing a similar risk of receiving healthcare for DUDs compared to Norwegian-born young people. However, young men from Afghanistan and the former Yugoslavia deviated from this pattern. This may be partially explained by the length of time spent in Norway. The results add support to previous qualitative studies suggesting that punitive drug policies may disproportionately affect men from minority groups. Further research controlling for parental household-level factors is warranted.**

## Background

Young refugees are part of a vulnerable subgroup whose condition of arrival varies considerably compared with other immigrants. Having fled due to the risk of violence or death, many arrive in their host countries with few available resources after having undertaken perilous migration journeys. As such, they often experience a wide range of mental health issues – especially young unaccompanied refugee minors who arrive alone with no family or adult companion to care for them [1,2]. Young refugees must also contend with several post-migration difficulties – such as social exclusion, discrimination and adjustment to a different cultural context – that can influence both their health and socioeconomic outcomes [2–4]. As a result of these challenges, they may be more vulnerable to problematic drug use as a way to cope, while simultaneously facing barriers in accessing psychiatric healthcare services [3,5–7].

In many Western countries, men from minority ethnic groups have disproportionately been on the receiving end of punitive drug policies [8–10]. Reports have shown that this is part of the daily reality experienced by men with an immigrant background in Scandinavian countries [11–15]. Experiences of discrimination have the potential to exacerbate other psychological burdens associated with life as a refugee. Research related to prejudicial policing and its psychological effects on young refugees is particularly timely because the Nordic countries are becoming more diverse, with immigrants and their Norwegian-born children now accounting for a substantial portion of the population [16].

An area of research that has not been fully explored is the utilisation of healthcare services related to drug use disorders (DUDs) and charges related to narcotics among young refugees. Given their increased vulnerability to mental health issues and potentially higher frequency of negative encounters with the police, this study aimed to investigate the patterns of psychiatric healthcare utilisation related to DUDs and criminal charges related to narcotics among the young refugee population in Norway compared with Norwegians born in Norway. As men encounter the police to a greater extent than women, and the young refugee population in Norway is diverse in terms of origin and condition of arrival (unaccompanied versus accompanied), we conducted sex-stratified analyses that accounted for these factors.

## Data and methods

### Study population

The term “young refugee” was used in this study to describe refugees who arrived in Norway before the age of 18 years. They were further divided into two groups based on the condition of their arrival because this represents a difference in the types of challenges and resources that might influence the outcomes of the study [2,4]: (a) accompanied refugee minors, who arrived with an adult parent/guardian as well as those who arrived to be reunited with a family member who had previously been granted asylum in Norway; and (b) unaccompanied refugee minors, who arrived alone with no adult legally responsible for them and had been granted residency based on their unaccompanied status. Municipalities receive a financial grant from the national government for resettling refugees. Unaccompanied refugee minors are often referred to the municipal Child Welfare Services (*Barnevernet*), which assumes responsibility for their housing and integration [4].

The analytical sample consisted of 15,068 young refugees and a comparison group of 573,241 Norwegians born in Norway to two Norwegian-born parents. All the young people were born between 1983 and 1994 and had registered residency in Norway as of 2015. The refugees’ countries of origin were collapsed into groups based on region, with the largest regions distinguished from each other – namely, states from the former Yugoslavia, the Horn of Africa and the Middle East. Afghanistan was treated as a separate group because refugees from this country represented a substantial portion of the sample, while the remaining countries of origin were subsumed into an “other” category (see Appendix 1, Supplementary material, available online, for the list of countries).

Norwegian population registers were used to investigate healthcare utilisation and criminal charges related to narcotics for the study population (see Appendix 2, Supplementary material, available online, for an overview of the registers used in the study). Norway collects administrative register data from different government agencies that can be linked using a unique personal number and anonymised for research purposes. In this study, register data from Statistics Norway’s population register provided sociodemographic information on the study population and the types of criminal offences based on records provided by the police. Data on immigration and condition of arrival were taken from the register of the Norwegian Directorate of Immigration. The project was approved by the Norwegian Data Protection Authority (17/00058-3/CDG).

## Outcome measures

The first outcome measure was based on having received healthcare services (both in- and outpatient care) due to DUDs taken from the Norwegian Patient Register. This outcome was based on the ICD-10 diagnostic codes related to mental and behavioural disorders due to psychoactive substance use (F11–F16, F18 and F19), as well as psychiatric services related to drug rehabilitation (Z50.3), drug abuse counselling and surveillance (Z71.5), drug use problems related to lifestyle (Z72.2) and poisoning by narcotics and psychodysleptic drugs with undetermined intent (Y12).

The two outcomes for criminal offences related to narcotics were taken from Statistics Norway’s 2015 classification based on a pre-structured set of codes used by the Norwegian police and other legal entities (STRASAK-codes). One outcome relates to narcotic offences based on the Medicines Act (*Legemiddelloven*), which includes the use of narcotics (code 6AAAZZ) and minor possession (code 6AABZZ). The other type of offence pertains to the production, transport, purchase, and sale or storage of narcotic substances under the Penal Code (*Straffeloven*) (code 6ABAZZ). Because the study is interested in personal consumption, aggravated drug charges punishable under the Penal Code (code 6ABBZZ), which involve large quantities of illegal substances with the possible intent for distribution and sale, were not included in this study. No distinction was made for the type of narcotics involved. The status of ‘person charged’ is given after the prosecuting authorities have deemed an individual as the perpetrator after an investigation and only charges that have received a decision with legal efficacy are included in the registers [17].

### Covariates

Information on the level of urbanicity of the municipality of residence (the domicile) was categorised using the Centrality Index from Statistics Norway. This index is a continuous variable that was averaged throughout the observation period, then categorised based on the 2018 cutoffs for the categorical version of the index – which range from 1 to 6, with 1 being the most central (urban) level. Because immigrants and refugees are concentrated in Norway’s largest cities, the most rural categories (4–6) were collapsed together. Centrality level 2 also included major cities other than Oslo and was thus combined with centrality level 1, leading to the final three categories used in the adjusted models: urban areas (levels 1 and 2), towns and suburbs (level 3) and rural districts (levels 4–6).

Additional covariates included birth year, which was grouped into three-year intervals starting in 1983, and completion of upper secondary education by age 21 years (dichotomised) to reflect educational attainment.

### Statistical analyses

Cox regression was used to estimate the hazard ratios (HR) with 95% confidence intervals (CI) to analyse the risk of experiencing the three outcomes of the study: psychiatric healthcare related to DUD and charges related to narcotics based on the Medicines Act and the Penal Code. Risk among the refugee populations was assessed relative to their Norwegian peers born in Norway in all analyses and stratified by sex. Person-time was calculated from their 21st birthday or 1 January 2008 (whichever came last) up to the 31 December 2015 (see Appendix 3, Supplemental material, available online). The start of the observation period allowed for the inclusion of unaccompanied refugee minors in the analysis because most of these refugees were around the ages of 15–17 years on arrival. This also provided a lag period of four years in the calculation of person-time to ensure that all individual refugees had had enough time to familiarise themselves with the healthcare system and other social services. Cases were censored upon emigration or death. The proportional hazards assumption was tested using the Schoenfeld residuals test (Appendices 4 and 5, Supplemental material, available online). Covariates that did not meet the proportional hazards assumption were controlled for by stratification. The variables for region of origin and condition of arrival for the male sample failed the proportional hazards assumption test for models pertaining to the Medicines Act. Because these were the main predictors of interest, an interaction term for time was added for the two models to deal with non-proportionality.

## Results

Table I presents descriptive statistics for the study population. The largest group of young refugees by origin were from the states in the former Yugoslavia (29.2%), followed by those coming from the Middle East (21.1%) and countries from the Horn of Africa (12.8%). Afghanistan was a particularly large group, comprising 15.4% of the total young refugee sample. However, male refugees from Afghanistan accounted for 22.6% of the total male sample, whereas female refugees from Afghanistan comprised only 5.8% of the total female sample. In terms of domicile, a substantial portion of both sexes lived in a mostly urban setting (57.5% for male refugees and 64.4% for female refugees), with only a small fraction in the most rural districts (15.6% for male refugees, and 10.8% for female refugees). Unaccompanied young refugees represented 22.9% of the refugee sample, with unaccompanied male refugees being more than three times the fraction of unaccompanied female refugees (32.1% and 10.6% respectively).

**Table I.** Descriptive statistics of the study populations.

	Afghanistan		Former Yugoslavia		Horn of Africa		Middle East		Other		Total refugees		Norway (reference)	
	(*N*=2325)		(*N*=4404)		(*N*=1925)		(*N*=3177)		(*N*=3237)		(*N*=15,068)		(*N*=573,241)	
	Male	Female	Male	Female	Male	Female	Male	Female	Male	Female	Male	Female	Male	Female
	(*N*=1951)	(*N*=374)	(*N*=2231)	(*N*=2173)	(*N*=939)	(*N*=986)	(*N*=1781)	(*N*=1396)	(*N*=1716)	(*N*=1521)	(*N*=8618)	(*N*=6450)	(*N*=289,225)	(*N*=284,016)
**Birth cohort**														
1983–1985	104 (5.3)	39 (10.4)	561 (25.1)	561 (25.8)	235 (25.0)	216 (21.9)	393 (22.1)	292 (20.9)	259 (15.1)	184 (12.1)	1552 (18.0)	1292 (20.0)	64,751 (22.4)	64,752 (22.8)
1986–1988	250 (12.8)	85 (22.7)	613 (27.5)	599 (27.6)	205 (21.8)	209 (21.2)	432 (24.3)	343 (24.6)	363 (21.2)	286 (18.8)	1863 (21.6)	1522 (23.6)	71,242 (24.6)	69,956 (24.6)
1989–1991	309 (15.8)	112 (29.9)	631 (28.3)	642 (29.5)	186 (19.8)	217 (22.0)	479 (26.9)	377 (27.0)	518 (30.2)	490 (32.2)	2123 (24.6)	1838 (28.5)	77,301 (26.7)	75,518 (26.6)
1992–1994	1288 (66.0)	138 (36.9)	426 (19.1)	371 (17.1)	313 (33.3)	344 (34.9)	477 (26.8)	384 (27.5)	576 (33.6)	561 (36.9)	3080 (35.7)	1798 (27.9)	75,931 (26.3)	73,790 (26.0)
**Domicile**														
Urban areas	897 (46.0)	252 (67.4)	1216 (54.5)	1306 (60.1)	632 (67.3)	678 (68.8)	1186 (66.6)	1015 (72.7)	1023 (59.6)	902 (59.3)	4954 (57.5)	4153 (64.4)	109,481 (37.9)	112,791 (39.7)
Towns and suburbs	550 (28.2)	86 (23.0)	696 (31.2)	616 (28.3)	197 (21.0)	211 (21.4)	445 (25.0)	294 (21.1)	434 (25.3)	394 (25.9)	2322 (26.9)	1601 (24.8)	84,130 (29.1)	82,753 (29.1)
Rural districts	504 (25.8)	36 (9.6)	319 (14.3)	251 (11.6)	110 (11.7)	97 (9.8)	150 (8.4)	87 (6.2)	259 (15.1)	225 (14.8)	1342 (15.6)	696 (10.8)	95,614 (33.1)	88,472 (31.2)
**Secondary education (age 21 years)**														
No secondary education	1740 (89.2)	203 (54.3)	1032 (46.3)	696 (32.0)	763 (81.3)	708 (71.8)	1216 (68.3)	694 (49.7)	1145 (66.7)	857 (56.3)	5896 (68.4)	3158 (49.0)	90,494 (31.3)	67,236 (23.7)
Completed	211 (10.8)	171 (45.7)	1199 (53.7)	1477 (68.0)	176 (18.7)	278 (28.2)	565 (31.7)	702 (50.3)	571 (33.3)	664 (43.7)	2722 (31.6)	3292 (51.0)	198,731 (68.7)	216,780 (76.3)
**Condition of arrival**														
Accompanied refugee	379 (19.4)	341 (91.2)	2224 (99.7)	2167 (99.7)	455 (48.5)	525 (53.2)	1434 (80.5)	1358 (97.3)	1361 (79.3)	1375 (90.4)	5853 (67.9)	5766 (89.4)	–	–
Unaccompanied refugee	1572 (80.6)	33 (8.8)	7 (0.3)	6 (0.3)	484 (51.5)	461 (46.8)	347 (19.5)	38 (2.7)	355 (20.7)	146 (9.6)	2765 (32.1)	684 (10.6)	–	–
**Years of residence**														
<8	1711 (87.7)	151 (40.4)	81 (3.6)	63 (2.9)	485 (51.7)	474 (48.1)	535 (30.0)	223 (16.0)	814 (47.4)	642 (42.2)	3626 (42.1)	1553 (24.1)	–	–
8–15	235 (12.0)	214 (57.2)	1276 (57.2)	1183 (54.4)	313 (33.3)	343 (34.8)	833 (46.8)	725 (51.9)	579 (33.7)	581 (38.2)	3236 (37.5)	3046 (47.2)	–	–
>15	5 (0.3)	9 (2.4)	874 (39.2)	927 (42.7)	141 (15.0)	169 (17.1)	413 (23.2)	448 (32.1)	323 (18.8)	298 (19.6)	1756 (20.4)	1851 (28.7)	–	–
**Age at immigration (years)**														
0–6	17 (0.9)	27 (7.2)	1092 (48.9)	1116 (51.4)	160 (17.0)	177 (18.0)	504 (28.3)	511 (36.6)	315 (18.4)	291 (19.1)	2088 (24.2)	2122 (32.9)	–	–
7–13	208 (10.7)	190 (50.8)	980 (43.9)	910 (41.9)	228 (24.3)	288 (29.2)	624 (35.0)	593 (42.5)	597 (34.8)	625 (41.1)	2637 (30.6)	2606 (40.4)	–	–
14–17	1726 (88.5)	157 (42.0)	159 (7.1)	147 (6.8)	551 (58.7)	521 (52.8)	653 (36.7)	292 (20.9)	804 (46.9)	605 (39.8)	3893 (45.2)	1722 (26.7)	–	–
**Censored**														
Died	6 (0.3)	1 (0.3%)	6 (0.3)	1 (0.0)	2 (0.2)	3 (0.3)	4 (0.2)	1 (0.1)	5 (0.3)	2 (0.1)	23 (0.3)	8 (0.1)	843 (0.3)	334 (0.1)
Emigrated	28 (1.4)	10 (2.7)	48 (2.2)	77 (3.5)	54 (5.8)	59 (6.0)	70 (3.9)	78 (5.6)	66 (3.8)	42 (2.8)	266 (3.1)	266 (4.1)	4082 (1.4)	6590 (2.3)
**Experienced outcome**														
Drug use disorder	13 (0.7)	0 (0)	36 (1.6)	14 (0.6)	24 (2.6)	12 (1.2)	30 (1.7)	18 (1.3)	25 (1.5)	9 (0.6)	128 (1.5)	53 (0.8)	3784 (1.3)	2527 (0.9)
Charged (Medicines Act)	45 (2.3)	0 (0)	96 (4.3)	11 (0.5)	75 (8.0)	8 (0.8)	109 (6.1)	9 (0.6)	97 (5.7)	5 (0.3)	422 (4.9)	33 (0.5)	6692 (2.3)	1706 (0.6)
Charged (Penal Code)	25 (1.3)	1 (0.3)	50 (2.2)	6 (0.3)	66 (7.0)	5 (0.5)	67 (3.8)	7 (0.5)	52 (3.0)	4 (0.3)	260 (3.0)	23 (0.4)	4094 (1.4)	835 (0.3)

Data presented as *n* (%).

Table II presents the unadjusted and adjusted HRs for healthcare utilisation for DUDs and narcotics charges among the refugee groups compared with young Norwegians born in Norway.

**Table II.** Healthcare due to drug use disorders and narcotic offences by country of origin.

	**Female**						**Male**					
	*Drug use disorder*						*Drug use disorder*					
	Unadjusted			Adjusted			Unadjusted			Adjusted		
	HR	95% CI	*p*	HR	95% CI	*p*	HR	95% CI	*p*	HR	95% CI	*p*
Norway (reference)	1			1			1			1		
Afghanistan^a^							0.82	0.48 – 1.42	0.48	**0.39**	**0.23 – 0.67**	**<0.001**
Former Yugoslavia	0.67	0.40–1.14	0.14	**0.49**	**0.29–0.83**	**<0.01**	1.15	0.83–1.60	0.40	0.83	0.60–1.16	0.28
Horn of Africa	1.50	0.85–2.65	0.16	0.58	0.33–1.03	0.06	**2.11**	**1.41–3.15**	**<0.001**	1.01	0.67–1.51	0.98
Middle East	1.49	0.94–2.37	0.09	0.75	0.47–1.19	0.22	1.31	0.92–1.88	0.14	0.70	0.49–1.00	0.05
Other	0.77	0.40–1.47	0.42	**0.39**	**0.20–0.75**	**<0.001**	1.25	0.84–1.85	0.26	0.69	0.47–1.03	0.07
	*Narcotic charges (Medicines Act)*						*Narcotic charges (Medicines Act)*					
	Unadjusted			Adjusted			Unadjusted			Adjusted		
	HR	95% CI	*p*	HR	95% CI	*p*	HR	95% CI	*p*	HR	95% CI	*p*
Norway (reference)	1			1			1			1		
Afghanistan^a^							**1.47**	**1.10–1.98**	**0.01**	**0.40**	**0.26–0.63**	**<0.001**
Former Yugoslavia	0.79	0.43–1.42	0.42	0.63	0.35–1.14	0.13	**1.77**	**1.44–2.16**	**<0.001**	1.32	0.99–1.76	0.06
Horn of Africa	1.48	0.74–2.96	0.27	0.64	0.32–1.28	0.21	**3.81**	**3.03–4.78**	**<0.001**	**1.56**	**1.10–2.22**	**0.01**
Middle East	1.10	0.57–2.11	0.78	0.62	0.32–1.20	0.16	**2.72**	**2.25–3.29**	**<0.001**	**1.37**	**1.04–1.81**	**0.03**
Other	0.63	0.26–1.51	0.30	**0.32**	**0.13–0.77**	**0.01**	**2.71**	**2.22–3.32**	**<0.001**	**1.35**	**1.01–1.80**	**0.04**
	*Narcotic charges (Medicines Act)*						*Narcotic charges (Medicines Act)*					
	Unadjusted						Adjusted					
	HR	95% CI	*p*	HR	95% CI	*p*	HR	95% CI	*p*	HR	95% CI	*p*
Norway (reference)	1			1			1			1		
Afghanistan	1.05	0.15–7.49	0.96	0.54	0.08–3.85	0.54	1.44	0.97–2.13	0.07	0.69	0.47–1.03	0.07
Former Yugoslavia	0.87	0.39–1.94	0.74	0.70	0.31–1.56	0.38	**1.48**	**1.12–1.96**	**0.01**	1.13	0.86–1.50	0.38
Horn of Africa	1.90	0.79–4.58	0.15	0.85	0.35–2.04	0.71	**5.50**	**4.32–7.02**	**<0.001**	**2.96**	**2.32–3.79**	**<0.001**
Middle East	1.75	0.83–3.69	0.14	1.01	0.48–2.13	0.98	**2.73**	**2.15–3.48**	**<0.001**	**1.59**	**1.25–2.03**	**<0.001**
Other	1.04	0.39–2.78	0.93	0.56	0.21–1.49	0.24	**2.41**	**1.83–3.16**	**<0.001**	**1.41**	**1.07–1.85**	**0.01**

HR: hazard ratio; CI: confidence interval.

Adjusted for birth cohort, domicile and completion of secondary education

a Cannot be estimated in the female group due to non-events.

For healthcare related to DUDs, female refugees did not show any risk difference compared with their Norwegian peers, apart from the lower risk observed among those from the former Yugoslavia (aHR = 0.49, 95% CI 0.29–0.83) and from the other regions (aHR = 0.39, 95% CI 0.20–0.75). Only male refugees from Afghanistan had significantly lower hazards when controls were added (aHR = 0.39, 95% CI 0.23–0.67). None of the other origin groups had significantly different risks compared with the reference group in both the unadjusted and adjusted models.

In terms of drug offences related to the Medicines Act, female refugees did not show any difference in the risk of being charged compared with female Norwegians, but the ‘others’ category had significantly lower hazards in the adjusted model (aHRs 0.32, 95% CI 0.13–0.77). For young male refugees, those coming from the former Yugoslavia had similar risk patterns compared with Norwegians, whereas those coming from Afghanistan had significantly lower hazards (aHRs 0.40, 95% CI 0.26–0.63). The remaining origin groups all had higher hazards of getting charged.

For drug offences based on the Penal Code, female refugees had similar hazards compared with female Norwegians. For young male refugees, however, only those from the former Yugoslavia and Afghanistan demonstrated similar risks compared with their Norwegian peers after applying controls, while the remaining origin groups all had higher hazards. Those a coming from the Horn of Africa had the highest hazard of being charged, almost three times higher than male Norwegians (aHR = 2.96; 95% CI 2.32–3.79).

Table III presents the results based on the refugees’ condition of arrival. Compared with Norwegians born in Norway, the risk for receiving care for DUDs was significantly lower for both accompanied and unaccompanied female refugees (aHR = 0.57, 95% CI 0.43–0.75; aHR = 0.09, 95% CI 0.09–0.66, respectively). Unaccompanied male refugees also had significantly lower hazards in the adjusted model (aHR = 0.45, 95% CI 0.31–0.66), whereas no significant difference was seen for accompanied male refugees in the adjusted model (aHR = 0.83, 95% CI 0.68–1.02).

**Table III.** Healthcare due to drug use disorders and drug offences by condition of arrival (accompanied and unaccompanied).

	**Female**						**Male**					
	*Drug use disorder*						*Drug use disorder*					
	Unadjusted			Adjusted			Unadjusted			Adjusted		
	HR	95% CI	*p*	HR	95% CI	*p*	HR	95% CI	*p*	HR	95% CI	*p*
Norway (reference)	1			1			1			1		
Accompanied	0.98	0.74–1.31	0.91	**0.57**	**0.43–0.75**	**<0.001**	**1.36**	**1.12–1.66**	**<0.001**	0.83	0.68–1.02	0.07
Unaccompanied	0.70	0.26–1.87	0.48	**0.25**	**0.09–0.66**	**0.01**	1.00	0.68–1.46	0.99	**0.45**	**0.31–0.66**	**<0.001**
	*Narcotic charges (Medicines Act)*						*Narcotic charges (Medicines Act)*					
	Unadjusted			Adjusted			Unadjusted			Adjusted		
	HR	95% CI	*p*	HR	95% CI	*p*	HR	95% CI	*p*	HR	95% CI	*p*
Norway (reference)	1			1			1			1		
Accompanied	0.95	0.67–1.35	0.77	**0.59**	**0.42–0.84**	**<0.001**	**2.56**	**2.30–2.86**	**<0.001**	**1.42**	**1.21–1.68**	**<0.001**
Unaccompanied	0.26	0.04–1.84	0.18	**0.10**	**0.01–0.72**	**0.02**	**1.75**	**1.42–2.16**	**<0.001**	**0.60**	**0.44–0.83**	**<0.001**
	*Narcotic charges (Penal Code)*						*Narcotic charges (Penal Code)*					
	Unadjusted			Adjusted			Unadjusted			Adjusted		
	HR	95% CI	*p*	HR	95% CI	*p*	HR	95% CI	*p*	HR	95% CI	*p*
Norway (reference)	1			1			1			1		
Accompanied	1.22	0.78–1.90	0.38	1.31	0.59–2.91	0.51	**2.48**	**2.15–2.86**	**<0.001**	**1.62**	**1.41–1.88**	**<0.001**
Unaccompanied	1.60	0.51–4.97	0.42	1.45	0.30–7.13	0.65	**2.12**	**1.65–2.72**	**<0.001**	1.03	0.80–1.33	0.79

HR: hazard ratio; CI: confidence interval.

Adjusted for birth cohort, domicile and completion of secondary education.

The results for charges related to the Medicines Act showed that both accompanied and unaccompanied female refugees had a lower risk of being charged than Norwegians born in Norway (aHR = 0.59, 95% CI 0.42–0.84; aHR = 0.10, 95% CI 0.01–0.72, respectively). For the male group, accompanied male refugees had an increased risk of being charged (aHR = 1.42; 95% CI 1.21–1.68) whereas unaccompanied male refugees had lower hazards (aHR = 0.60, 95% CI 0.44–0.83).

For more serious charges related to the Penal Code, no difference in the risk pattern was observed in the female refugee sample. However, accompanied male refugees again had higher hazards (aHR = 1.62; 95% CI 1.41–1.88), whereas no difference in risk was observed when comparing unaccompanied male refugees to Norwegians born in Norway (aHR = 1.03, 95% CI 0.80–1.33)

## Discussion

This register-based study aimed to explore the patterns of healthcare utilisation based on DUDs and charges related to narcotics among the young refugee population in Norway. The results showed a clear sex difference in the risks for being charged for a narcotic offence and healthcare utilisation for DUDs, whereby female refugees demonstrated an equal or lower risk of experiencing these outcomes than their Norwegian counterparts. Taken together, the observed risk patterns for both DUDs and charges related to narcotics correspond well with previous research showing the self-reported consumption of illicit substances to be lower for young women [18–21].

Young male refugees generally showed a higher risk of being charged for narcotic offences, but exhibited no difference in their use of healthcare for DUDs compared with Norwegians born in Norway. However, two origin groups deviated from this pattern: those from Afghanistan, for whom a lower risk was found for charges related to the Medicines Act and healthcare utilisation for DUDs, and those from the former Yugoslavia, who showed no risk differential in all outcomes of the study. The results for those coming from the former Yugoslavia, mostly refugees from Bosnia-Herzegovina, are consistent with previous reports showing outcomes across several integration indicators to be more similar to Norwegians born in Norway (e.g. level of education and labour market participation) [22]. This has been attributed to their swift resettlement, their cultural similarity with the majority population and collective residency granted by the Norwegian government [22], all of which may facilitate integration.

The risk patterns for these two groups may also be influenced by their length of residency and age of arrival in Norway. Studies examining similar outcomes have indicated that, over time, rates of substance use among young refugees tend to align with those of their peers born in Norway [7,20]. Most of the refugees from the former Yugoslavia arrived in the 1990s and, as such, have been in Norway longer than the other groups in this study. By contrast, a pattern of lower risk can be seen in the male refugees from Afghanistan who arrived more recently and at a much older age (around 88.5% arriving at ages 14–17 years), with 80.6% of them being unaccompanied minors. The results for region of origin are also reflected in the results based on the condition of arrival, where unaccompanied young refugees showed a pattern of lower hazards for DUDs and charges related to the Medicine Act than accompanied refugees.

Previous monitors have shown that illicit substance use is higher among young ethnic Norwegians [21,23]. However, our findings indicated little or no difference in the use of specialised healthcare for DUDs, while charges related to narcotics were generally higher in the male refugee population. This discrepancy indicates a form of social inequality between Norwegians born in Norway and a subgroup within the immigrant population. This may lend support to the claim that ethnic minority men may have been disproportionately affected by prejudicial practices in policing. Although prejudice cannot be inferred from register data alone, our findings are in line with qualitative studies carried out in Scandinavia in which men with a non-Western immigrant background reported being detained more often than their white counterparts [11–15].

The intersection of location, immigrant background and social class may partially explain the numbers we see. Reports on adolescent substance use in Oslo found that young people living in the affluent, western side of the city report a higher use of illicit substances, but it is often their peers living in the more diverse, eastern parts who get into trouble more often [21,23,24]. Ethnically diverse neighbourhoods tend to attract a higher police presence, which could lead to more stop-and-search encounters and, as a consequence, higher rates of arrests [8,13,25–27]. These encounters may increase the likelihood of being charged with an offence and could lead to a criminal conviction (or, in some cases, incarceration) in young adulthood, setting them on a path of marginalisation that could alter economic/labour market opportunities later in life [28,29]. The Attorney General of Norway published a report in 2022 that highlighted regulatory uncertainties regarding the use of coercive measures by the Norwegian police in minor drug incidents. In certain cases, these measures were used by individual officers without consultation from police prosecutors [30].

The lower healthcare utilisation for DUDs among young refugees, especially for recently arrived groups (unaccompanied refugees and those coming from Afghanistan), may indicate a different pattern of healthcare utilisation related to the short time they have spent in Norway, while post-migration contextual factors (e.g., prejudicial policing) may explain the higher hazards for narcotic-related offences for young male refugees in general.

This study’s strength lies in utilising high-quality national registers that cover the entire population of interest and allow for analyses that may not be available elsewhere. Nevertheless, several limitations need to be highlighted. First, the Norwegian Patient Register only captures DUDs in those who have been in contact with the healthcare system and have received an official diagnosis. As this is an indicator of healthcare utilisation, there may be people who face barriers in accessing healthcare who are not captured in the registers. As a result, the true proportion of young male refugees suffering from DUDs in Norway may be higher than reported in our study. Second, the refugee population in Norway is fairly heterogenous, arriving from different regions of the world, under different immigration policy frameworks and with varying lengths of time spent in Norway. These factors may influence multiple health and integration outcomes that limit the generalizability of our findings. Third, there were some limitations in the number of covariates included in the analyses due to availability of data. Household income and parental socioeconomic status at the start of the observation period could not be controlled for and further studies are needed to investigate how these factors may influence the examined associations.

## Conclusions

This register-based study showed that disparities in the utilisation of healthcare related to DUDs and narcotic-related charges varied by sex, country of origin and condition of arrival. Our findings suggest that male refugees in general are at a higher risk of being charged with narcotic offences than their Norwegian-born peers. Post-migration factors, as well as the time spent in the host country, may be important factors that influence the risk of experiencing the outcomes assessed in this study. Future research should explore the role of parental socioeconomic status, as well as integration policies and police reforms, to further our understanding of social factors that may impact healthcare use for DUDs as well as narcotic offences in this young immigrant population.

## Supplemental Material

sj-xlsx-1-sjp-10.1177_14034948231201895 – Supplemental material for Narcotic offences and drug use disorders among young refugees in Norway Supplemental material, sj-xlsx-1-sjp-10.1177_14034948231201895 for Narcotic offences and drug use disorders among young refugees in Norway by Ryan T. Europa, Ketil Eide, Anders Hjern, Helio Manhica and Andrea Dunlavy in Scandinavian Journal of Public Health

sj-xlsx-2-sjp-10.1177_14034948231201895 – Supplemental material for Narcotic offences and drug use disorders among young refugees in Norway Supplemental material, sj-xlsx-2-sjp-10.1177_14034948231201895 for Narcotic offences and drug use disorders among young refugees in Norway by Ryan T. Europa, Ketil Eide, Anders Hjern, Helio Manhica and Andrea Dunlavy in Scandinavian Journal of Public Health

sj-xlsx-3-sjp-10.1177_14034948231201895 – Supplemental material for Narcotic offences and drug use disorders among young refugees in Norway Supplemental material, sj-xlsx-3-sjp-10.1177_14034948231201895 for Narcotic offences and drug use disorders among young refugees in Norway by Ryan T. Europa, Ketil Eide, Anders Hjern, Helio Manhica and Andrea Dunlavy in Scandinavian Journal of Public Health

sj-xlsx-4-sjp-10.1177_14034948231201895 – Supplemental material for Narcotic offences and drug use disorders among young refugees in Norway Supplemental material, sj-xlsx-4-sjp-10.1177_14034948231201895 for Narcotic offences and drug use disorders among young refugees in Norway by Ryan T. Europa, Ketil Eide, Anders Hjern, Helio Manhica and Andrea Dunlavy in Scandinavian Journal of Public Health

sj-xlsx-5-sjp-10.1177_14034948231201895 – Supplemental material for Narcotic offences and drug use disorders among young refugees in Norway Supplemental material, sj-xlsx-5-sjp-10.1177_14034948231201895 for Narcotic offences and drug use disorders among young refugees in Norway by Ryan T. Europa, Ketil Eide, Anders Hjern, Helio Manhica and Andrea Dunlavy in Scandinavian Journal of Public Health

## References

[1] Kale E Hjelde KH. Mental health challenges of immigrants in Norway, www.nakmi.no (2017, accessed 11 June 2022).

[2] Borsch AS de Montgomery CJ Gauffin K , et al. Health, education and employment outcomes in young refugees in the Nordic countries: A systematic review. Scand J Public Health 2019;47:735–47. 10.1177/1403494818787099 30067129

[3] Dunlavy A Gauffin K Berg L , et al. Health outcomes in young adulthood among former child refugees in Denmark, Norway and Sweden: A cross-country comparative study. Scand J Public Health 2021;51:330–8. 10.1177/14034948211031408 PMC10251467 34304618

[4] Lidén H Stang EG Eide K. The gap between legal protection, good intentions and political restrictions. Unaccompanied minors in Norway. Soc Work Soc 2017;15:1–20.

[5] Byqvist S. Drug-abusing women in Sweden: Marginalization, social exclusion and gender differences. J Psychoactive Drugs 2006;38:427–40. 10.1080/02791072.2006.10400582 17373559

[bibr6-14034948231201895] Ivsins A Yake K. Looking beyond harm: Meaning and purpose of substance use in the lives of marginalized people who use drugs. Drugs Educ Prev Policy 2020;27:27–36.

[7] Ivert AK Magnusson MM. Drug use and criminality among unaccompanied refugee minors: A review of the literature. Int J Migrat Health Soc Care 2020;16:93–107.

[8] Beckett K Nyrop K Pfingst L. Race, drugs, and policing: Understanding disparities in drug delivery arrests. Criminology 2006;44:105–37.

[9] Rosino ML Hughey MW. The war on drugs, racial meanings, and structural racism: A holistic and reproductive approach. Am J Econ Sociol 2018;77:849–92.

[10] Earp BD Lewis J Hart CL. Racial justice requires ending the war on drugs. Am J Bioeth. Epub ahead of print 2020;21:4–19. 33413050 10.1080/15265161.2020.1861364

[11] Sollund R. Racialisation in police stop and search practice – the Norwegian case. Crit Criminol 2006;14:265–92.

[bibr12-14034948231201895] Sollund R. Canteen banter or racism: is there a relationship between Oslo police’s use of derogatory terms and their attitudes and conduct towards ethnic minorities? J Scand Stud Criminol Crime Prev 2007;8:77–96.

[bibr13-14034948231201895] Solhjell R Saarikkomäki E Haller MB , et al. “We are seen as a threat”: Police stops of young ethnic minorities in the Nordic countries. Crit Criminol 2018;27:347–61.

[bibr14-14034948231201895] Saarikkomäki E Birk Haller M Solhjell R , et al. Suspected or protected? Perceptions of procedural justice in ethnic minority youth’s descriptions of police relations. Polic Soc 2021;31:386–401.

[15] Wästerfors D Burcar Alm V. ‘They are harsher to me than to my friend who is blonde’. Police critique among ethnic minority youth in Sweden. J Youth Stud 2020;23:170–88.

[16] Nordic Statistics. CITI03: Population by reporting country, country background, time, population category, sex and age, https://pxweb.nordicstatistics.org/pxweb/en/Nordic%20Statistics/Nordic%20Statistics__Integration%20and%20migration__Population/CITI03.px/ (2021, accessed 11 June 2022).

[17] Statistics Norway. Offences investigated 2017, https://www.ssb.no/en/sosiale-forhold-og-kriminalitet/kriminalitet-og-rettsvesen/statistikk/etterforskede-lovbrudd (2018, accessed 15 June 2022).

[18] Syse VL Brekke M Grimsrud MM , et al. Gender differences in acute recreational drug toxicity: A case series from Oslo, Norway. BMC Emerg Med 2019;19:29. 31035940 10.1186/s12873-019-0244-3 PMC6489220

[bibr19-14034948231201895] Heradstveit O Skogen JC Edland-Gryt M , et al. Self-reported illicit drug use among Norwegian university and college students. Associations with age, gender, and geography. Front Psychiatry 2020;11:1424. 10.3389/fpsyt.2020.543507 PMC7758438 33362594

[bibr20-14034948231201895] Manhica H Gauffin K Almqvist YB , et al. Hospital admission and criminality associated with substance misuse in young refugees – a Swedish national cohort study. PloS One 2016;11:e0166066. 10.1371/journal.pone.0166066 PMC5130257 27902694

[21] Bakken A. Ung I Oslo 2018, https://hdl.handle.net/20.500.12199/5133 (2018, accessed 11 June 2022).

[22] Dzamarija MT. Bosnians – the integration winners? Samfunnsspeilet 2016;4:15–20.

[23] Bakken A. Ung i Oslo 2023. Ungdomsskole og videregående skole, https://hdl.handle.net/11250/3065089 (2023, accessed 15 May 2023).

[24] Acharki F. Flere unge blir tatt for cannabis på østkanten – selv om det røykes mer i vest, https://www.nrk.no/osloogviken/flere-unge-blir-tatt-for-cannabis-pa-ostkanten-_-selv-om-det-roykes-mer-i-vest-1.14112100 (2018, accessed 11 June 2022).

[25] Staver AB Brekke J-P Søholt S. Scandinavia’s segregated cities–policies, strategies and ideals, https://hdl.handle.net/20.500.12199/1311 (2019, accessed 10 June 2022).

[26] Gaston S. Enforcing race: A neighborhood-level explanation of Black–White differences in drug arrests. Crime Delinq 2019;65:499–526.

[27] Manhica H Straatmann VS Lundin A , et al. Association between poverty exposure during childhood and adolescence, and drug use disorders and drug-related crimes later in life. Addiction 2021;116:1747–56. 10.1111/add.15336 PMC8247994 33197093

[28] Sandberg S. Black drug dealers in a White welfare state: Cannabis dealing and street capital in Norway. Br J Criminol 2008; 48: 604–619.

[29] Pedersen W Skardhamar T. Cannabis and crime: Findings from a longitudinal study. Addiction 2010;105:109–18. 10.1111/j.1360-0443.2009.02719.x 19839964

[30] Den Høyere Påtalemyndighet. Tvangsmiddelbruk i mindre alvorlige narkotikasaker, https://www.riksadvokaten.no/document/rapport-om-politiets-tvangsmiddelbruk/ (2022, accessed 15 May 2023).

